# Total synthesis of (−)-epimyrtine by a gold-catalyzed hydroamination approach

**DOI:** 10.3762/bjoc.9.242

**Published:** 2013-10-09

**Authors:** Thi Thanh Huyen Trinh, Khanh Hung Nguyen, Patricia de Aguiar Amaral, Nicolas Gouault

**Affiliations:** 1 Equipe PNSCM, UMR6226, Université de Rennes 1, 2 avenue du Pr Léon Bernard, 35043 Rennes Cedex, France

**Keywords:** epimyrtine, gold, gold catalysis, heterocycles, hydroamination, quinolizidine alkaloid, total synthesis

## Abstract

A new approach to the total synthesis of (−)-epimyrtine has been developed from D-alanine. The key step to access the enantiopure pyridone intermediate was achieved by a gold-mediated cyclization. Finally, various transformations afforded the natural product in a few steps and good overall yield.

## Findings

(−)-Epimyrtine, isolated from *Vaccinium myrtillus* (Ericaceae) [1–2], is a quinolizidine alkaloid. This alkaloid family exhibits potential pharmacological properties such as anticancer, antibacterial, antiviral and anti-inflammation activities [3–5]. This alkaloid has been a target of interest for synthetic chemists because of its structural simplicity among the family of quinolizidine structures. Since it has been isolated, numerous total syntheses of this alkaloid in racemic form have been reported in the literature. However, only a few asymmetric syntheses of (−)-epimyrtine have been described to date including the intramolecular allylsilane *N*-acyliminium ion cyclization [6], the organocatalytic aza-Michael reaction [7], the intramolecular Mannich reaction [8], and the iminium ion cascade reaction [9–10]. More efficient, convenient and highly stereoselective synthetic routes are still being sought after. In the past decades, gold catalysis has emerged as an important tool in a plethora of fields of synthetic organic chemistry, and after methodological investigations [11–16], the good functional group compatibility of gold catalysts renders gold catalysis a straightforward protocol in the realm of the synthesis of natural products [17–18].

**Scheme 1:**
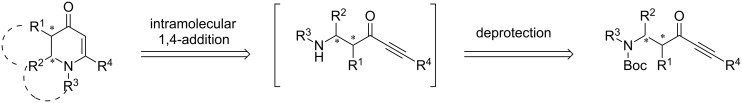
Previously reported approach from β-aminoynones for the synthesis of pyridones.

Herein we report a short total synthesis of (−)-epimyrtine employing an alternative strategy by using a gold(I)-catalyzed hydroamination of a β-aminoynone as the key step. Actually, cyclization of enantiopure α and β-aminoynones was successfully used in our group to access pyrrolidinone and pyridone heterocycles via a gold-mediated approach [19–20]. The use of β-aminoynone intermediates for the synthesis of 2,3-dihydropyridones was recently developed by Georg [21] (Scheme 1). This strategy involves the in situ deprotection of the amine function to permit the cyclization by Michael addition. However, in some instances partial racemization of the reaction products was observed [22].

Here, one major advantage of gold catalysis is the use of very mild conditions for the cyclization, thereby avoiding any racemization and obtaining N-protected compounds which may be useful for further transformations. In order to illustrate the efficiency of our method, we were interested in extending this methodology to quinolizidine privileged structures.

**Scheme 2:**
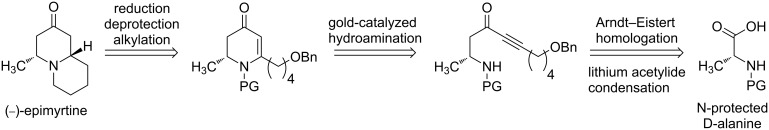
Retrosynthetic analysis of (−)-epimyrtine.

Our retrosynthetic analysis is shown in Scheme 2. We expected that the good side-chain functionality tolerance of the gold catalyst could easily provide chiral dihydropyridone from the corresponding β-aminoynone in a 6-*endo*-*dig* selective cyclization process. The β-aminoynone could be stereoselectively prepared in two steps from *N*-Boc-D-alanine.

**Scheme 3:**
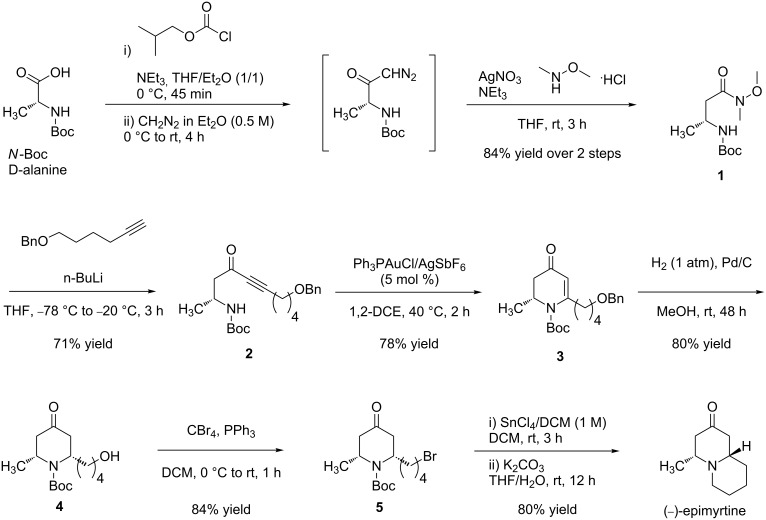
Synthesis of (−)-epimyrtine.

Preparation of the β-aminoynone **2** began with the Arndt–Eistert homologation [23] of *N*-Boc-protected D-alanine (Scheme 3). Thus, the *N*-Boc-D-alanine was treated with isobutyl chloroformate at 0 °C in THF/diethyl ether followed by the addition of diazomethane to afford the corresponding diazoketone. The Wolff rearrangement was then carried out by using silver nitrate in THF to give the intermediate ketene which was trapped with *N*,*O*-dimethylhydroxylamine to provide the corresponding Weinreb amide **1** in 84% yield over two steps. In the next step, the Weinreb amide **1** was added to a solution of O-protected 1-hexynol lithium acetylide to furnish the β-aminoynone **2** with a yield of 71%. With this key building block in hand, efforts were directed toward the gold-mediated intramolecular hydroamination for the construction of the chiral pyridone intermediate **3**. For this, PPh_3_AuSbF_6_ generated in situ from a 5 mol % mixture of PPh_3_AuCl and AgSbF_6_, in 1,2-dichloroethane afforded the desired pyridone **3**. These conditions, selected in our previous work, represent a good compromise in terms of reaction time, yield and cost of the catalyst. As an example, lower catalyst loading (1 mol %) does not affect the yield but lowers the reaction speed of the cyclization. A 5-*exo-dig* product was not observed, presumably as a result of electronic strain. The reaction was completed in 2 hours at 40 °C to afford **3** in good yield (78%).

Reduction and deprotection of **3** by means of H_2_ (1 atm) in the presence of Pd/C for 48 h afforded stereospecifically the corresponding piperidone **4** in 80% yield. Subsequent bromination with CBr_4_ in the presence of PPh_3_ (Appel reaction) gave **5** in 84% yield. Finally, deprotection with 1 M SnCl_4_ in dichloromethane then neutralization by using K_2_CO_3_ afforded the final product (−)-epimyritine in a 80% yield.

*N*-Cbz compound was also tested. In this case, the cyclization occurred in similar conditions as described for the N-Boc-protected compound and resulted in a good yield (81%). Yet, under the catalytic hydrogenolytic conditions as described above (H_2_, 1 atm, 10% Pd/C, rt, 48 h) only deprotection of the nitrogen occured, while the desired benzyl ether cleavage and pyridine reduction were unsuccessful. Increase of the pressure to 5 atm or replacement of Pd/C with Pd(OH)_2_ (Pearlman’s catalyst) did also not result in the obtainment of the desired product.

The natural product (−)-epimyritine was thus obtained over 6 steps in a 25% overall yield starting from *N*-Boc-D-alanine. The spectroscopic data and optical rotation are in agreement with the literature [6].

## Conclusion

In conclusion, we have achieved the asymmetric total synthesis of (−)- epimyrtine in six steps and with a good overall yield. We have demonstrated in this work that this natural product is easily accessible from D-alanine by a gold-mediated intramolecular hydroamination in a unique 6-*endo-dig* process. The approach provides a straightforward tool for synthetic applications toward quinolizidines and indolizidines.

## Experimental

All reagents of high quality were purchased from commercial suppliers and used without further puriﬁcation. All reactions requiring anhydrous conditions were performed under an argon atmosphere by using oven-dried glassware. 1,2-DCE and THF were distilled from CaH_2_ and Na/benzophenone, respectively. ^1^H and ^13^C NMR were recorded at 500 or 300 and 125 or 75 MHz respectively, by using CDCl_3_ (and TMS as internal standard). Chemical shifts, δ values are given in parts per million (ppm), coupling constants (*J*) are given in Hertz (Hz), and multiplicity of signals are reported as follows: s, singlet; d, doublet; t, triplet; q, quartet; quint, quintet; m, multiplet; app, apparent. Thin-layer chromatography was performed by using pre-coated silica gel plates (0.2 mm thickness).


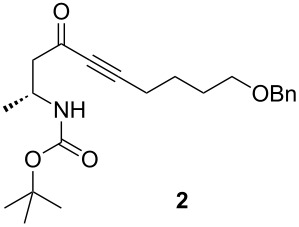


To a solution of 6-benzyloxyhex-1-yne (2.44 g, 12.9 mmol, 4 equiv) in 10 mL of dry THF was added dropwise a solution of *n*-BuLi (5 mL, 12.5 mmol, 3.8 equiv) at −78 °C under argon atmosphere. The reaction mixture was stirred for 45 min at −78 °C. Then, a solution of Weinreb amide **1** (800 mg, 3.3 mmol, 1 equiv) in 8 mL of dry THF was added dropwise at −78 °C. The reaction was stirred for 1 h. The reaction was warmed to −20 °C and stirred for 2 h. The reaction was quenched with a solution of 1 M NaH_2_PO_4_ (50 mL). The aqueous layer was extracted with ethyl acetate. The organic layer was washed with brine, dried over Na_2_SO_4_, concentrated under reduced pressure, and purified by silica gel column chromatography by using petroleum ether/ethyl acetate (9:1) as an eluent to give pure **2** as a yellow oil (250 mg, 71%). *R*_f_ 0.3 (petroleum ether/ethyl acetate, 8:2). [α]_D_^25^ +2.3 (*c* 1.7, CHCl_3_); ^1^H NMR (500 MHz, CDCl_3_) δ 1.20 (d, *J* = 6.8 Hz, 3H), 1.43 (s, 9H), 1.69–1.74 (m, 4H), 2.40 (t, *J* = 6.7 Hz, 2H), 2.66 (ABX system, *J*_AB_ = 16.2 Hz, *J*_BX_ = 6.2 Hz, 1H), 2.80 (ABX system, *J*_AB_ = 16.2 Hz, *J*_AX_ = 5.3 Hz, 1H), 3.50 (t, *J* = 5.8 Hz, 2H), 4.05–4.14 (m, 1H), 4.50 (s, 2H), 4.74 (brs, 1H), 7.29–7.35 (m, 5H); ^13^C NMR (125 MHz, CDCl_3_) δ 186.0, 155.0, 138.3, 128.3, 127.6, 127.5, 106.4, 94.8, 81.1, 72.9, 69.4, 51.3, 43.3, 28.8, 28.3, 24.5, 18.7; HRMS–ESI^+^ (*m*/*z*): [M + Na]^+^ calcd for C_22_H_31_NO_4_Na, 396.2151; found, 396.2153.


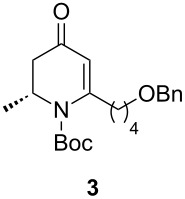


To a solution of **2** (838 mg, 2.2 mmol, 1 equiv) in 15 mL of anhydrous 1,2-dichloroethane was added PPh_3_AuCl (55 mg, 0.11 mmol, 5 mol %) and AgSbF_6_ (38 mg, 0.11 mmol, 5 mol %) under argon atmosphere. The mixture was stirred for 2 h at 40 °C. The reaction was cooled to room temperature and diluted with ether. The organic phase was filtered through a pad of Celite^®^, concentrated under reduced pressure, purified by silica gel column chromatography, and eluted with petroleum ether/ethyl acetate (9:1) to give **3** as a yellow oil (78%). *R*_f_ 0.25 (petroleum ether/ethyl acetate, 8:2). [α]_D_^25^ −226.9 (*c* 1.6, CHCl_3_); ^1^H NMR (500 MHz, CDCl_3_) δ 1.25 (d, *J* = 6.8 Hz, 3H), 1.52 (s, 9H), 1.55–1.67 (m, 4H), 2.23 (dt, *J* = 16.9 Hz, *J* = 1.5 Hz, 1H), 2.31 (ddd, *J* = 14.7 Hz, *J* = 8.5 Hz, *J* = 6.0 Hz, 1H), 2.81 (dd, *J* = 16.9 Hz, *J* = 6.2 Hz, 1H), 3.07 (ddd, *J* = 14.6 Hz, *J* = 9.3 Hz, *J* = 5.2 Hz, 1H), 3.47 (t, *J* = 6.2 Hz, 2H), 4.49 (s, 2H), 4.78 (app quintd, *J* = 6.6 Hz, *J* = 1.4 Hz, 1H), 5.36 (s, 1H), 7.30–7.36 (m, 5H); ^13^C NMR (125 MHz, CDCl_3_) δ 193.7, 158.3, 152.1, 138.5, 128.4, 127.6, 127.6, 111.1, 82.9, 72.9, 69.8, 52.1, 42.7, 35.8, 29.4, 28.1, 24.7, 16.5; HRMS–ESI^+^ (*m*/*z*): [M + Na]^+^ calcd for C_22_H_31_NO_4_Na, 396.2151; found, 396.2153.


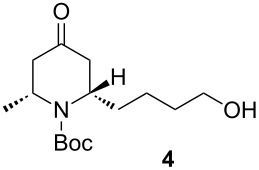


Pd/C (45 wt %) was added to a solution of **3** (585.7 mg, 1.57 mmol, 1 equiv) in 12 mL of MeOH, and the mixture was stirred under hydrogen atmosphere (1 atm) for 48 h at room temperature. The mixture was filtered through a pad of celite^®^, concentrated and purified by silica gel column chromatography eluting with petroleum ether/ethyl acetate to give **4** as a yellow oil (360 mg, 80%). *R*_f_ 0.25 (petroleum ether/ethyl acetate, 5:5). [α]_D_^25^ −21.9 (*c* 1.5, CHCl_3_); ^1^H NMR (500 MHz, CDCl_3_) δ 1.28 (d, *J* = 5.0 Hz, 3H), 1.35–1.42 (m, 3H), 1.49 (s, 9H), 1.57–1.62 (m, 4H) 2.28 (ddd, *J* = 14.9 Hz, *J* = 3.8 Hz, *J* = 1.6 Hz, 1H), 2.34 (dt, *J* = 15.0 Hz, *J* = 1.8 Hz, 1H), 2.68 (dd, *J* = 15.0 Hz, *J* = 7.6 Hz, 1H), 2.72 (dd, *J* = 14.9 Hz, *J* = 7.5 Hz, 1H), 3.64 (brs, 2H), 4.61–4.62 (m, 1H), 4.70 (brs, 1H); ^13^C NMR (125 MHz, CDCl_3_) δ 208.7, 154.9, 80.3, 62.6, 52.5, 48.4, 45.5, 43.8, 36.6, 32.2, 28.4, 23.0, 22.7; HRMS–ESI^+^ (*m*/*z*): [M + Na]^+^ calcd for C_15_H_27_NO_4_Na, 308.1838, found, 308.1838.


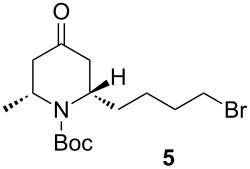


CBr_4_ (674.5 mg, 2 mmol, 2 equiv) and PPh_3_ (586.8 mg, 2.2 mmol, 2.2 equiv) were added to a solution of **4** (290 mg, 1 mmol, 1 equiv) in 6 mL of dichloromethane at 0 °C. The mixture was stirred at 0 °C for 15 min. Then, the reaction was warmed to room temperature and stirred for 1 h. The solvent was removed under reduced pressure and the mixture was purified by silica gel column chromatography by using dichloromethane/ethyl acetate (95:5) as eluent to give **5** as a yellow oil (293 mg, 84%). *R*_f_ 0.6 (dichloromethane/ethyl acetate, 9:1). [α]_D_^25^ −19.1 (*c* 1.1, CHCl_3_); ^1^H NMR (500 MHz, CDCl_3_) δ 1.27 (d, *J* = 7.0 Hz, 3H), 1.47–1.56 (m, 12H), 1.61–1.67 (m, 1H), 1.83–1.91 (m, 2H), 2.28 (ddd, *J* = 14.8 Hz, *J* = 3.5 Hz, *J* = 1.5 Hz, 1H), 2.32 (dt, *J* = 15.9 Hz, *J* = 1.7 Hz, 1H), 2.69 (dd, *J* = 15.3 Hz, *J* = 7.7 Hz, 1H), 2.73 (dd, *J* = 15.1 Hz, *J* = 7.9 Hz, 1H), 3.40 (td, *J* = 6.6 Hz, *J* = 1.5 Hz, 2H), 4.60–4.63 (m, 1H), 4.73 (brs, 1H); ^13^C NMR (125 MHz, CDCl_3_) δ 208.5, 154.8, 80.4, 52.4, 48.5, 45.5, 43.9, 36.1, 33.5, 32.2, 28.4, 25.5, 22.7; HRMS–ESI^+^ (*m*/*z*): [M + Na]^+^ calcd for C_15_H_26_NO_3_BrNa, 370.0994; found, 370.0990.


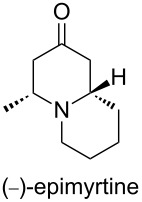


To a solution of **5** (263 mg, 0.75 mmol, 1 equiv) in 5 mL of CH_2_Cl_2_ under argon atmosphere was added a solution of 1 M SnCl_4_ in CH_2_Cl_2_ (3.8 mL, 5.0 equiv). The reaction was stirred at room temperature for 3 h. The solvent was removed under reduced pressure. Then 25 mL of THF and 40 mL of an aqueous saturated solution of K_2_CO_3_ were added. The mixture was stirred for 12 h at room temperature. The aqueous phase was extracted with CH_2_Cl_2_, the combined organic layers were washed with brine, dried over Na_2_SO_4_, and concentrated in vacuo. The crude residue was purified by silica gel column chromatography eluting with dichloromethane/MeOH (9:1) to give (−)-epimyrtine as a yellow oil (102 mg, yield 80%). *R*_f_ 0.3 (dichloromethane/MeOH, 9:1). [α]_D_^25^ −16.9 (*c* 1.2, CHCl_3_); ^1^H NMR (500 MHz, CDCl_3_) δ 1.20 (d, *J* = 5.6 Hz, 3H), 1.23–1.32 (m, 1H), 1.39–1.45 (m, 1H), 1.59–1.69 (m, 2H), 1.70–1.78 (m, 2H), 1.83 (td, *J* = 11.2 Hz, *J* = 2.1 Hz, 1H), 2.17 (brt, *J* = 11.1 Hz, 1H), 2.22–2.32 (m, 2H), 2.27–2.32 (m, 2H), 2.34–2.45 (m, 3H), 3.32 (d, *J* = 11.2 Hz, 1H); ^13^C NMR (125 MHz, CDCl_3_) δ 208.4, 62.0, 59.3, 51.0, 49.8, 48.7, 34.2, 25.9, 23.9, 20.7; HRMS–ASAP(*m*/*z*): [M + H]^+^ calcd for C_10_H_18_NO, 168.1388; found, 168.1387.

## Supporting Information

File 1 Spectra of new compounds.
