# Supplementary material for: Total synthesis of (−)-epimyrtine by a gold-catalyzed hydroamination approach
Source: Beilstein J Org Chem. 2013 Oct 9;9:2042–7. doi: 10.3762/bjoc.9.242 (PMC3817515; doi:10.3762/bjoc.9.242)
Supplement: File 1 — Spectra of new compounds. [file Beilstein_J_Org_Chem-09-2042-s001.pdf]

Supporting Information

for

Total synthesis of (−)-epimyrtine by a gold-catalyzed hydroamination approach

Thi Thanh Huyen Trinh, Khanh Hung Nguyen, Patricia de Aguiar Amaral and Nicolas Gouault*

Address: Equipe PNSCM, UMR6226, Université de Rennes 1, 2 avenue du Pr Léon Bernard, 35043 Rennes Cedex, France

Email: Nicolas Gouault - nicolas.gouault@univ-rennes1.fr

* Corresponding author

Spectra of new compounds

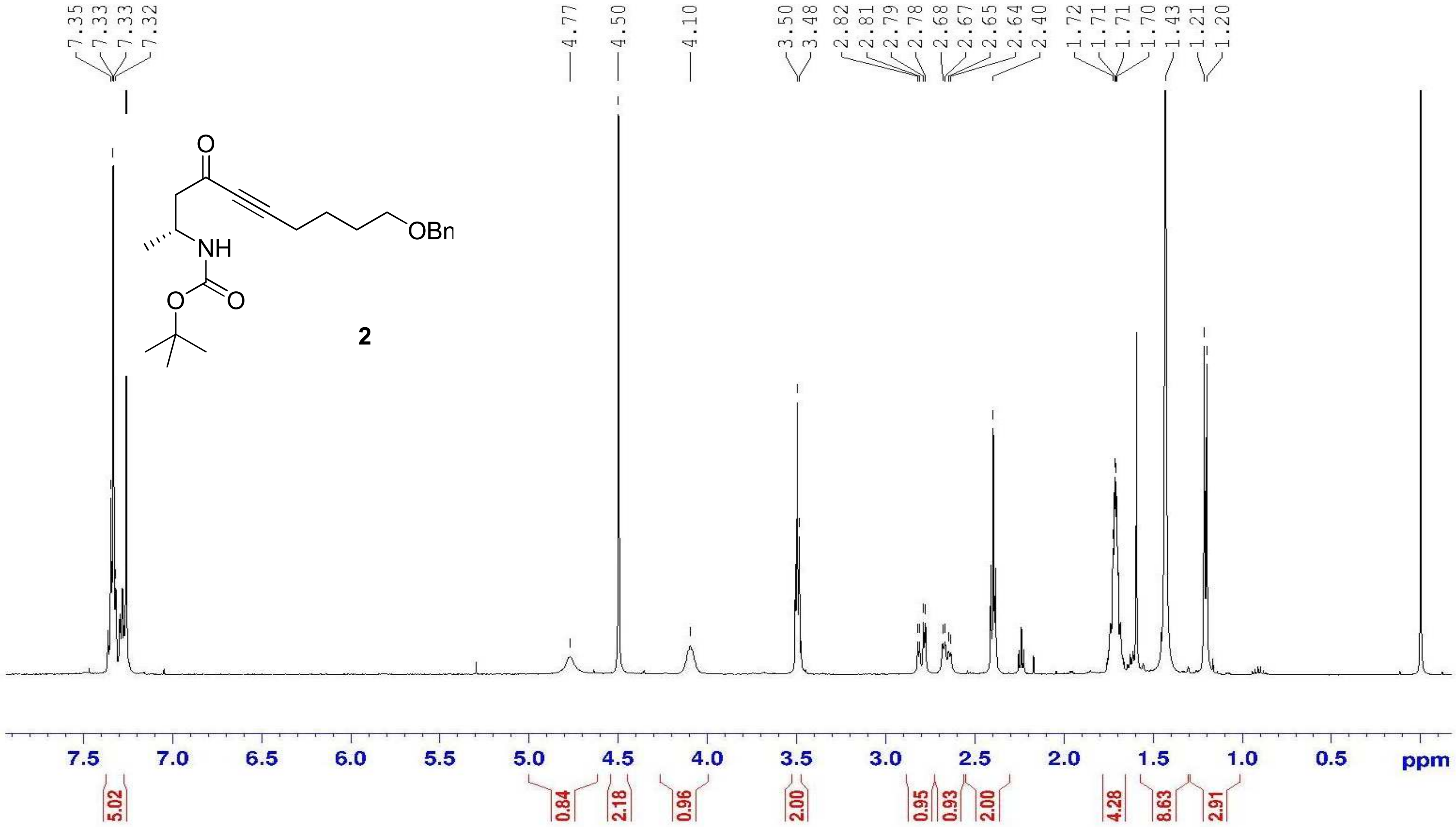
7.35 7.33 7.33 7.32
4.77
4.50
4.10
3.50 3.48
2.82 2.81 2.79 2.78 2.68 2.67 2.65 2.64 2.40
1.72 1.71 1.71 1.70 1.43 1.21 1.20
O
NH
O
O
OBn
2
7.5 7.0 6.5 6.0 5.5 5.0 4.5 4.0 3.5 3.0 2.5 2.0 1.5 1.0 0.5 ppm
5.02
0.84
2.18
0.96
2.00
0.95
0.93
2.00
4.28
8.63
2.91

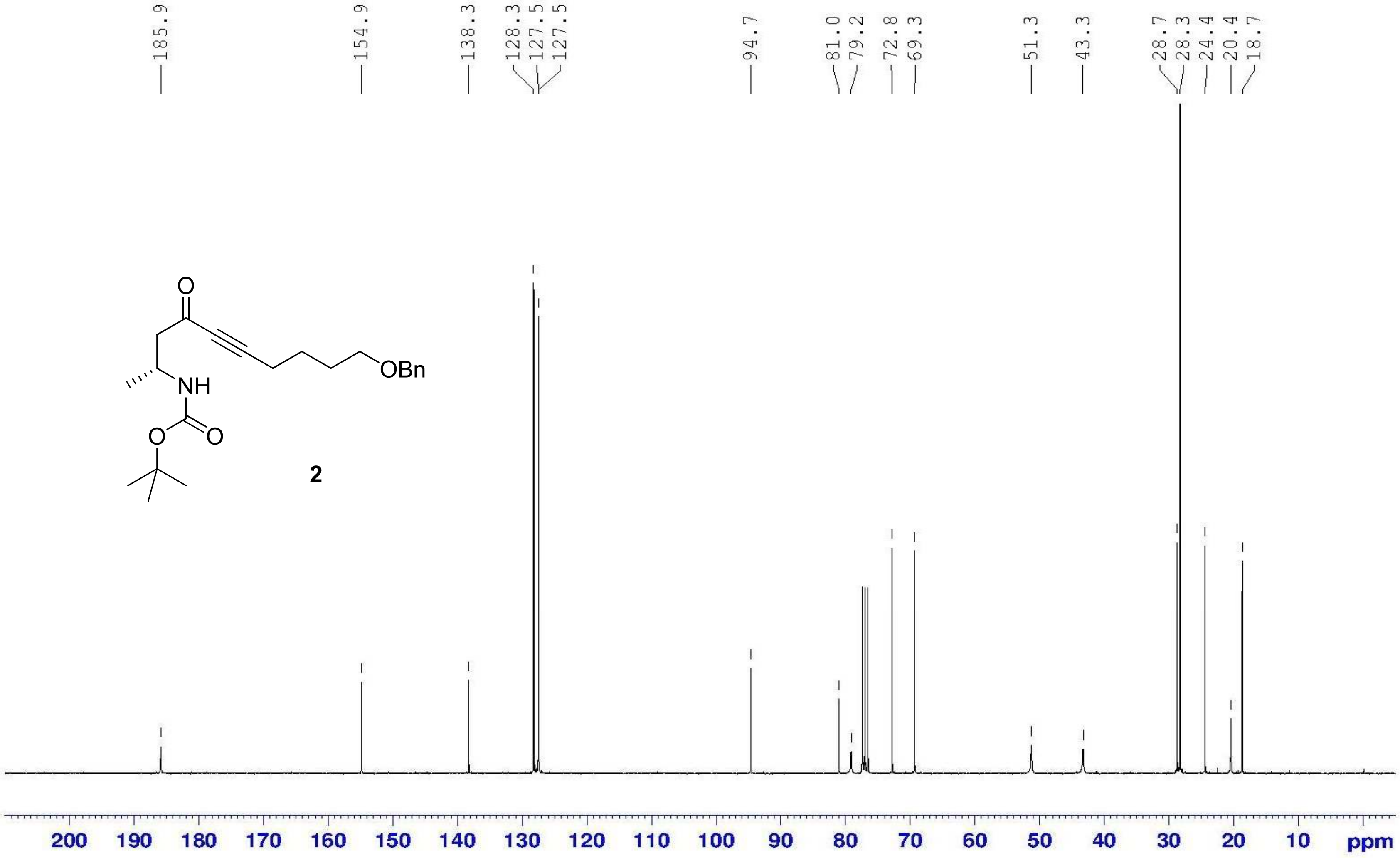
185.9
154.9
138.3
128.3
127.5
127.5
94.7
81.0
79.2
72.8
69.3
51.3
43.3
28.7
28.3
24.4
20.4
18.7
O
NH
O
O
OBn
2
200 190 180 170 160 150 140 130 120 110 100 90 80 70 60 50 40 30 20 10 ppm

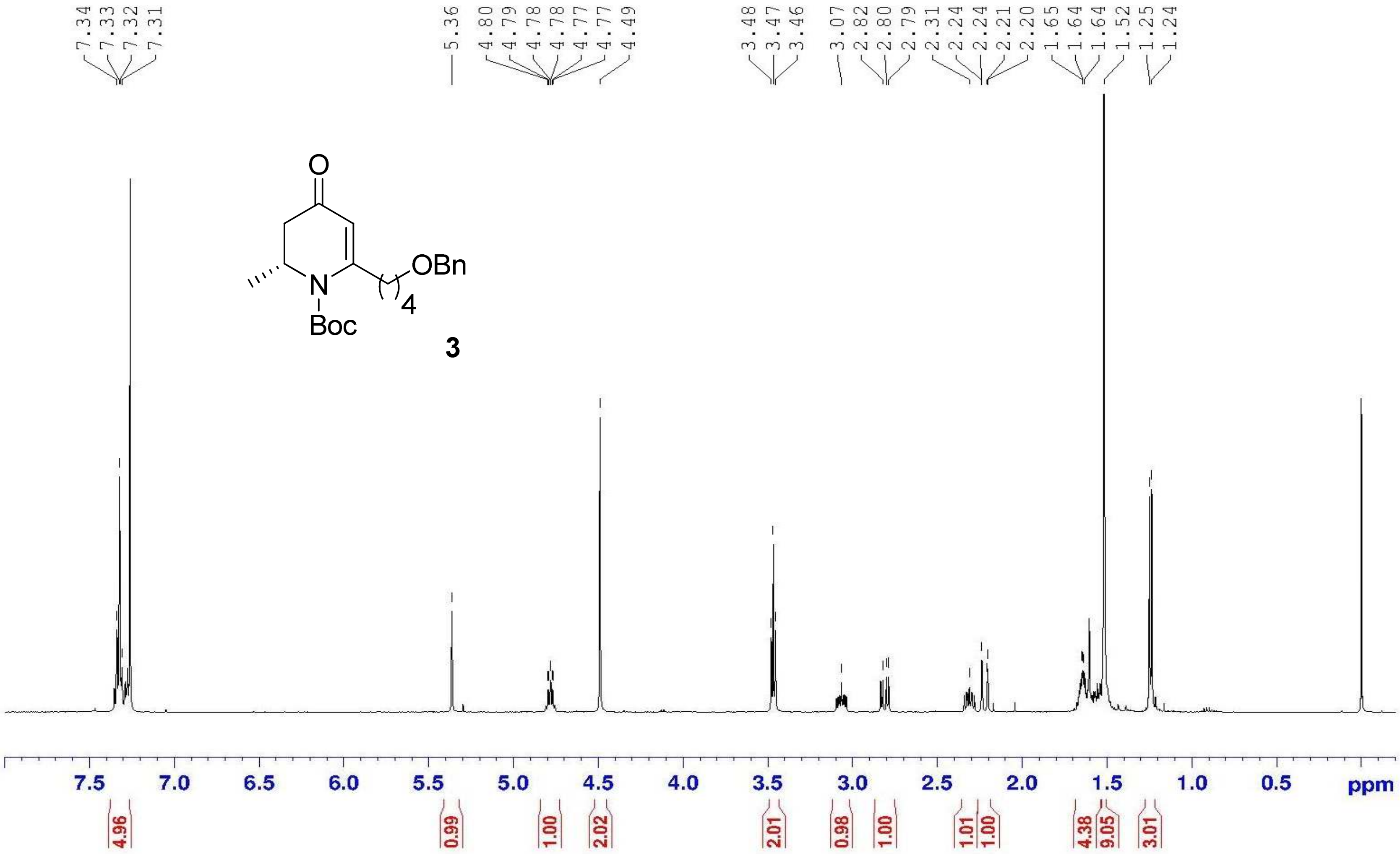
7.34 7.33 7.32 7.31
5.36
4.80 4.79 4.78 4.78 4.77 4.77 4.49
3.48 3.47 3.46
3.07 2.82 2.80 2.79
2.31 2.24 2.24 2.21 2.20
1.65 1.64 1.64 1.52 1.25 1.24
O
OBn
N
4
Boc
3
7.5 7.0 6.5 6.0 5.5 5.0 4.5 4.0 3.5 3.0 2.5 2.0 1.5 1.0 0.5 ppm
4.96 0.99 1.00 2.02 2.01 0.98 1.00 1.01 1.00 4.38 9.05 3.01

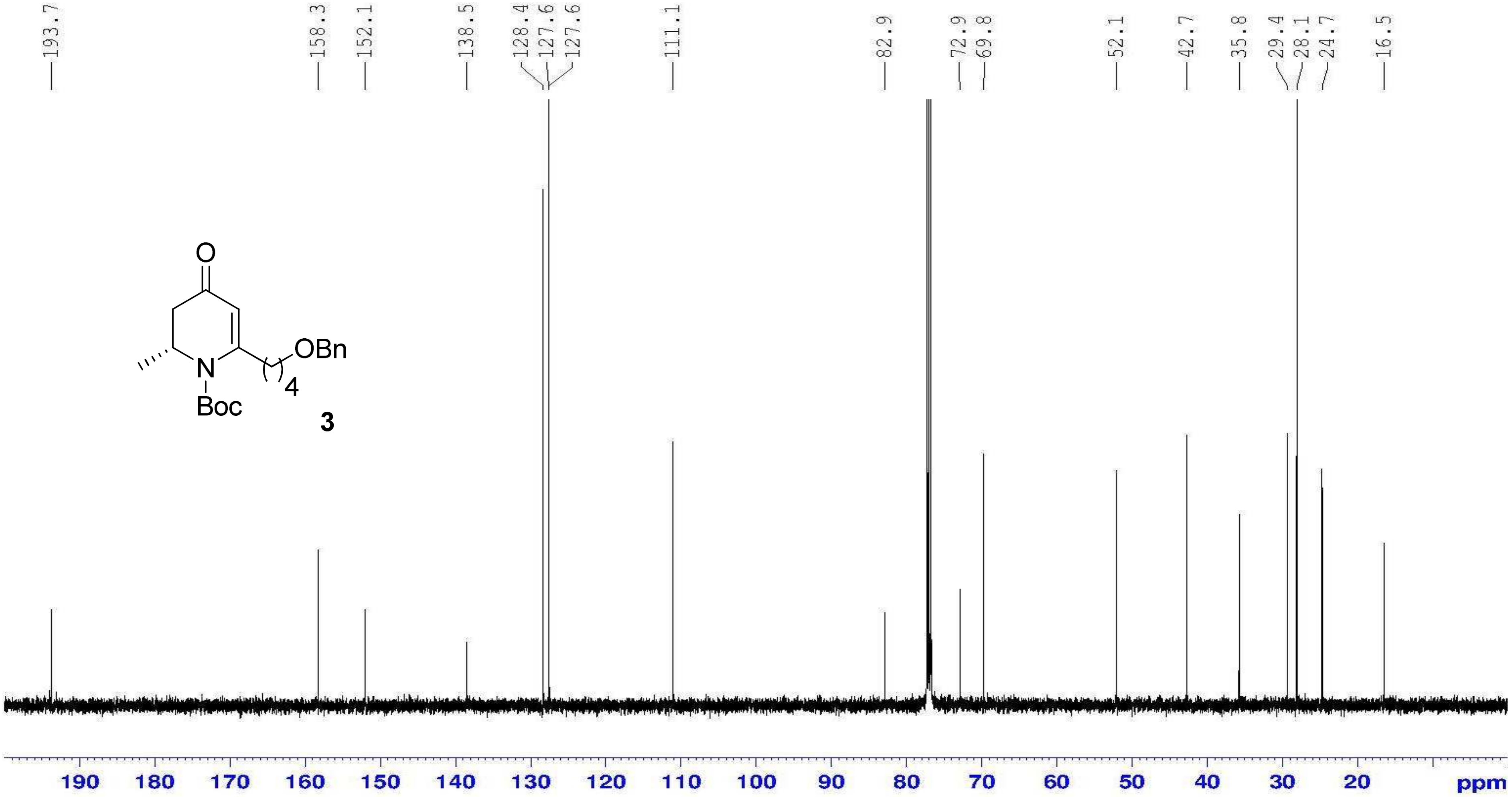
193.7
158.3
152.1
138.5
128.4
127.6
127.6
111.1
82.9
72.9
69.8
52.1
42.7
35.8
29.4
28.1
24.7
16.5
O
N
Boc
OBn
4
3
190 180 170 160 150 140 130 120 110 100 90 80 70 60 50 40 30 20
ppm

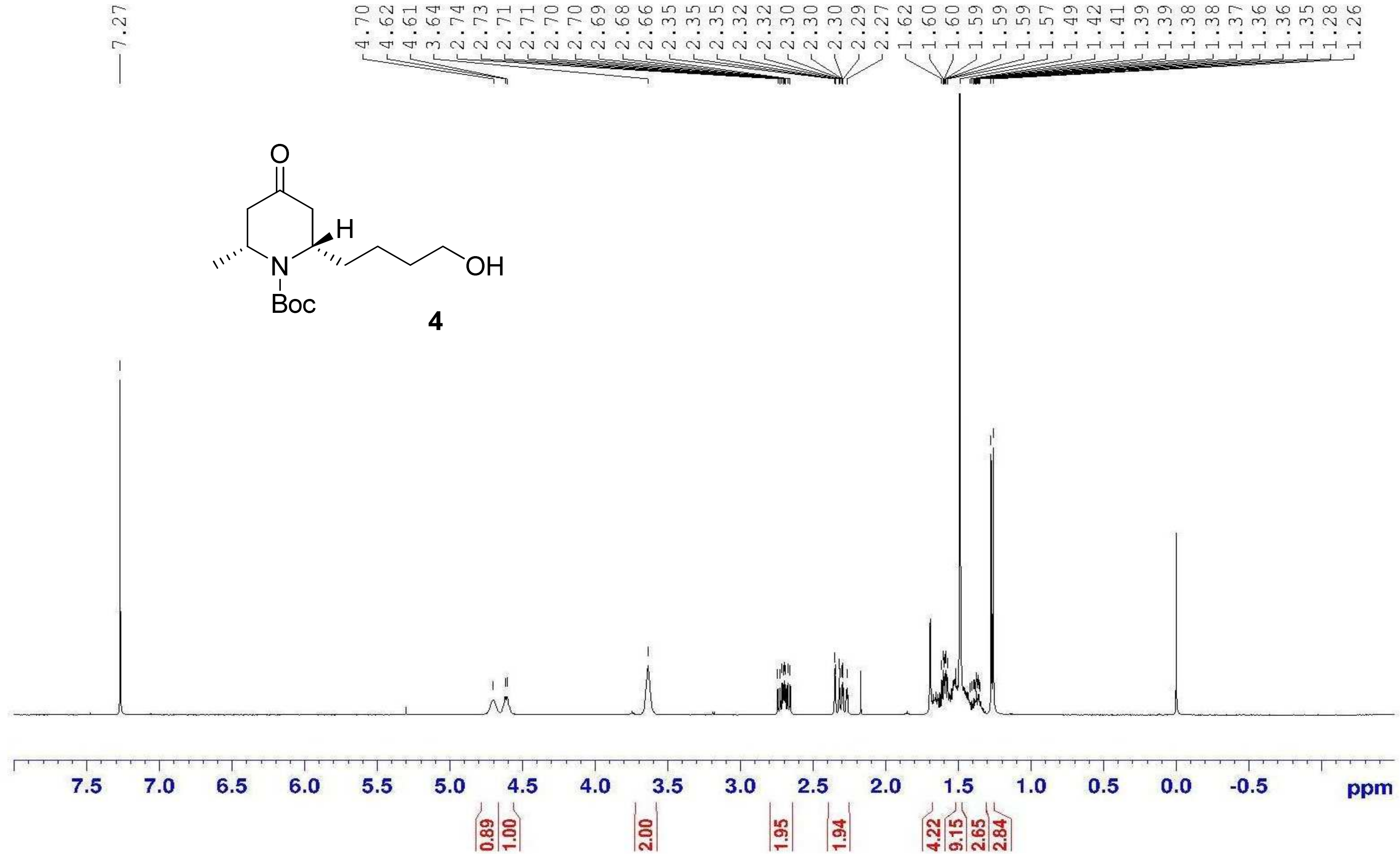
7.27
4.70 4.62 4.61 3.64 2.74 2.73 2.71 2.71 2.70 2.70 2.69 2.68 2.66 2.35 2.35 2.35 2.32 2.32 2.30 2.30 2.30 2.29 2.27 1.62 1.60 1.60 1.59 1.59 1.59 1.57 1.49 1.42 1.41 1.39 1.39 1.38 1.38 1.37 1.36 1.36 1.35 1.28 1.26
O
H
N
OH
Boc
4
7.5 7.0 6.5 6.0 5.5 5.0 4.5 4.0 3.5 3.0 2.5 2.0 1.5 1.0 0.5 0.0 -0.5 ppm
0.89 1.00 2.00 1.95 1.94 4.22 9.15 2.65 2.84

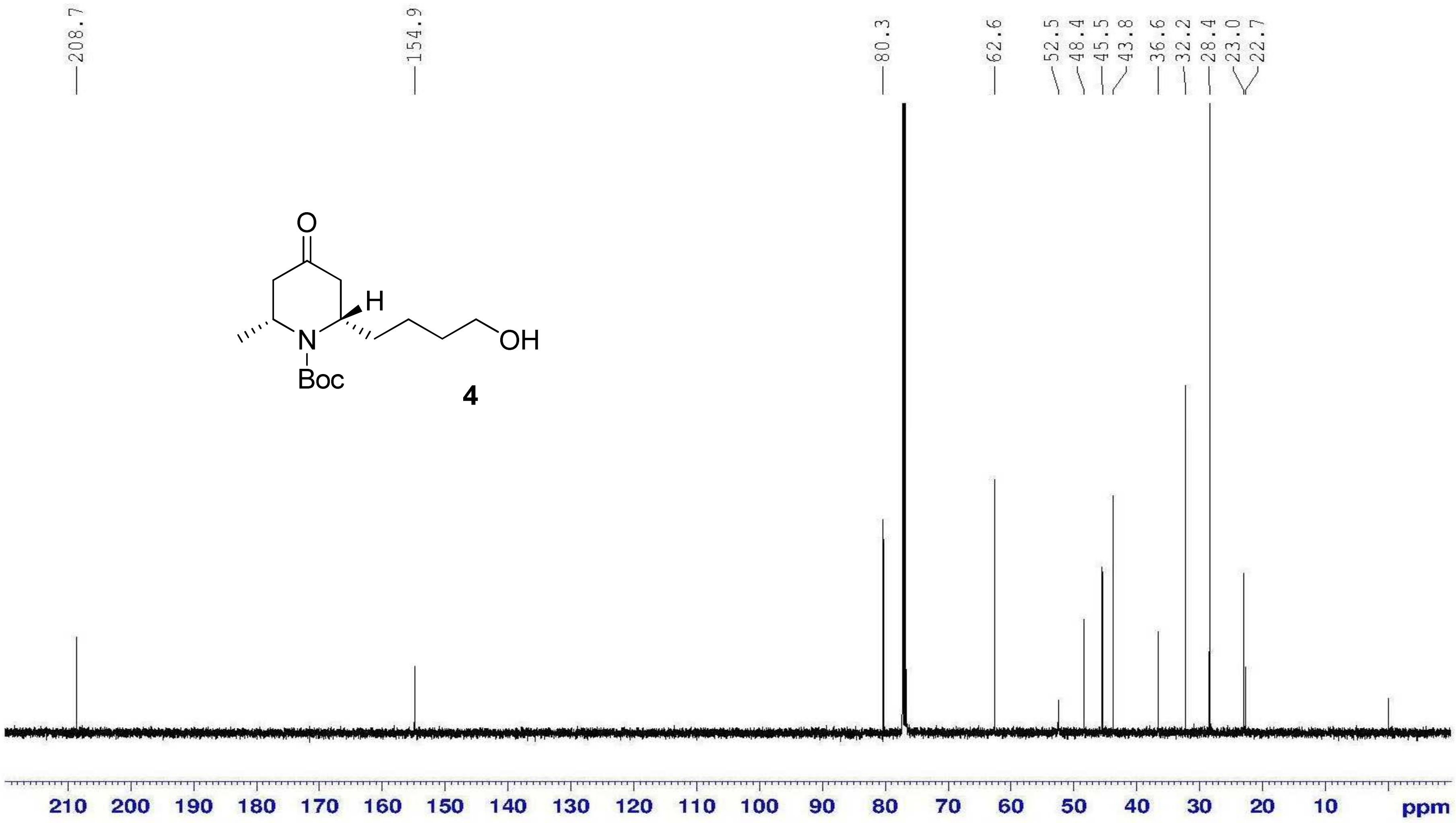
208.7
154.9
80.3
62.6
52.5
48.4
45.5
43.8
36.6
32.2
28.4
23.0
22.7
O
H
N
OH
Boc
4
210 200 190 180 170 160 150 140 130 120 110 100 90 80 70 60 50 40 30 20 10 ppm

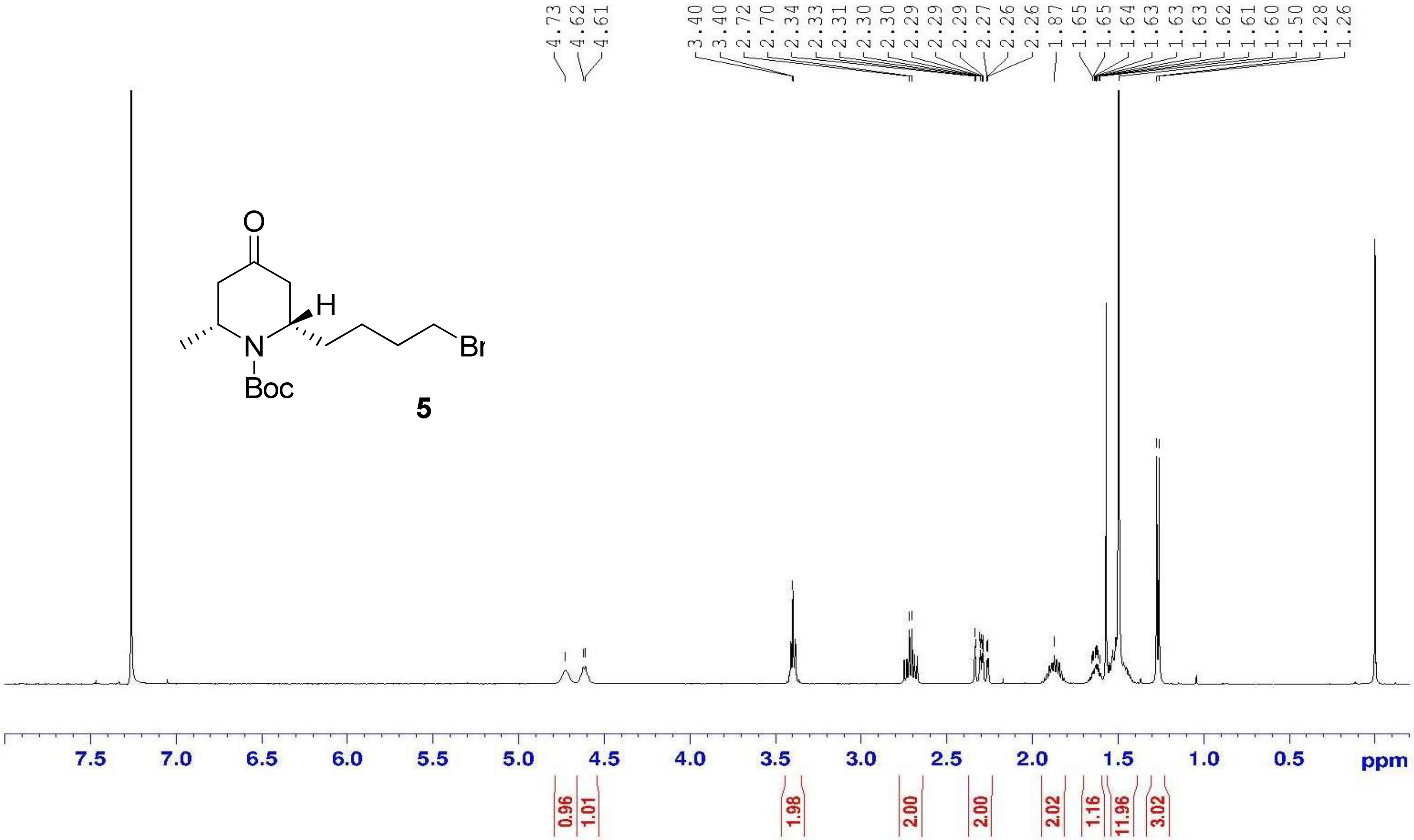
5
4.73
4.62
4.61
3.40
3.40
2.72
2.70
2.34
2.33
2.31
2.30
2.30
2.29
2.29
2.29
2.27
2.26
2.26
1.87
1.65
1.65
1.64
1.63
1.63
1.63
1.62
1.61
1.60
1.50
1.28
1.26
7.5
7.0
6.5
6.0
5.5
5.0
4.5
4.0
3.5
3.0
2.5
2.0
1.5
1.0
0.5
ppm
0.96
1.01
1.98
2.00
2.00
2.02
1.16
11.96
3.02

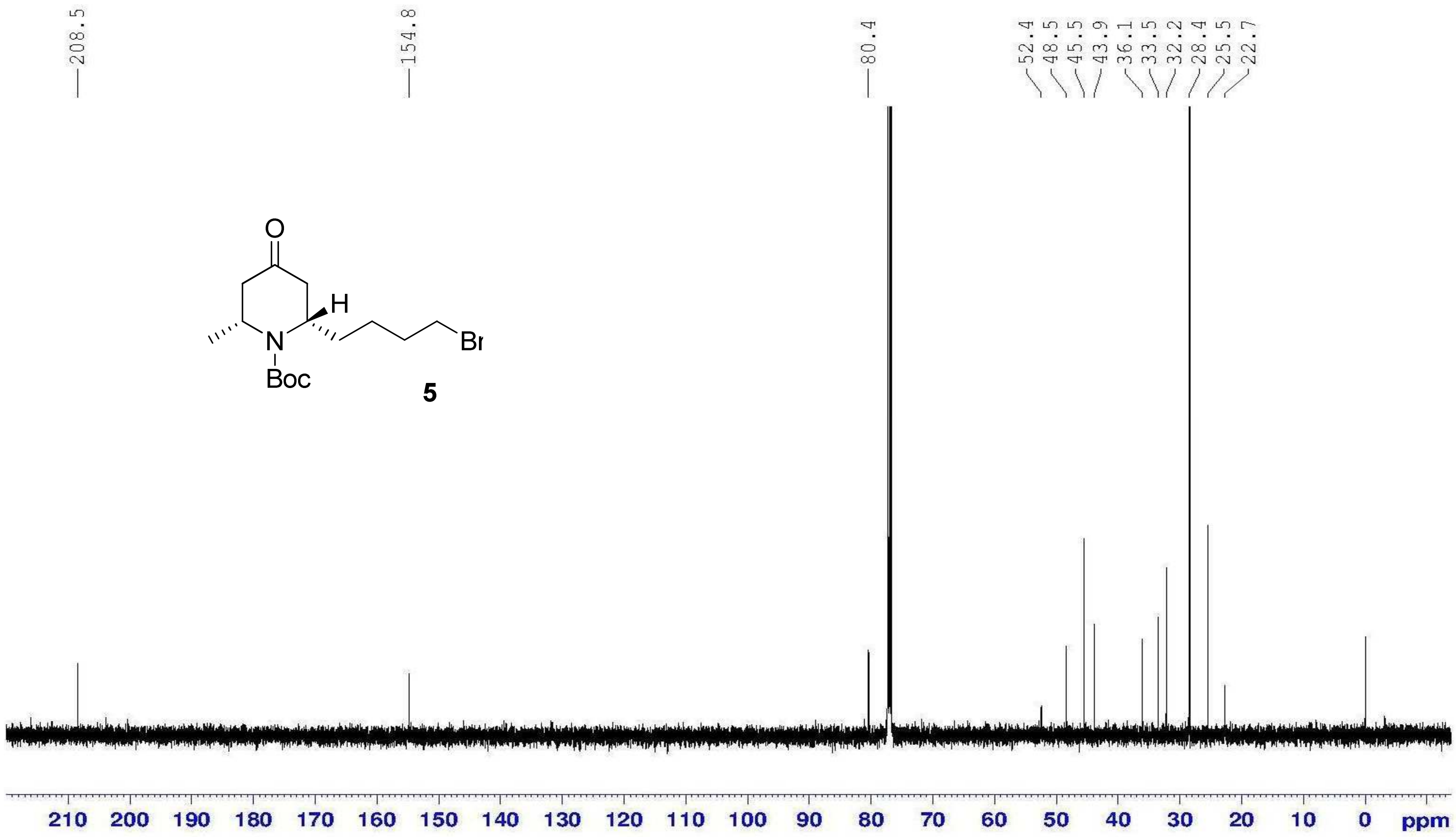
208.5
154.8
80.4
52.4
48.5
45.5
43.9
36.1
33.5
32.2
28.4
25.5
22.7
O
H
N
Boc
Br
5
210 200 190 180 170 160 150 140 130 120 110 100 90 80 70 60 50 40 30 20 10 0 ppm

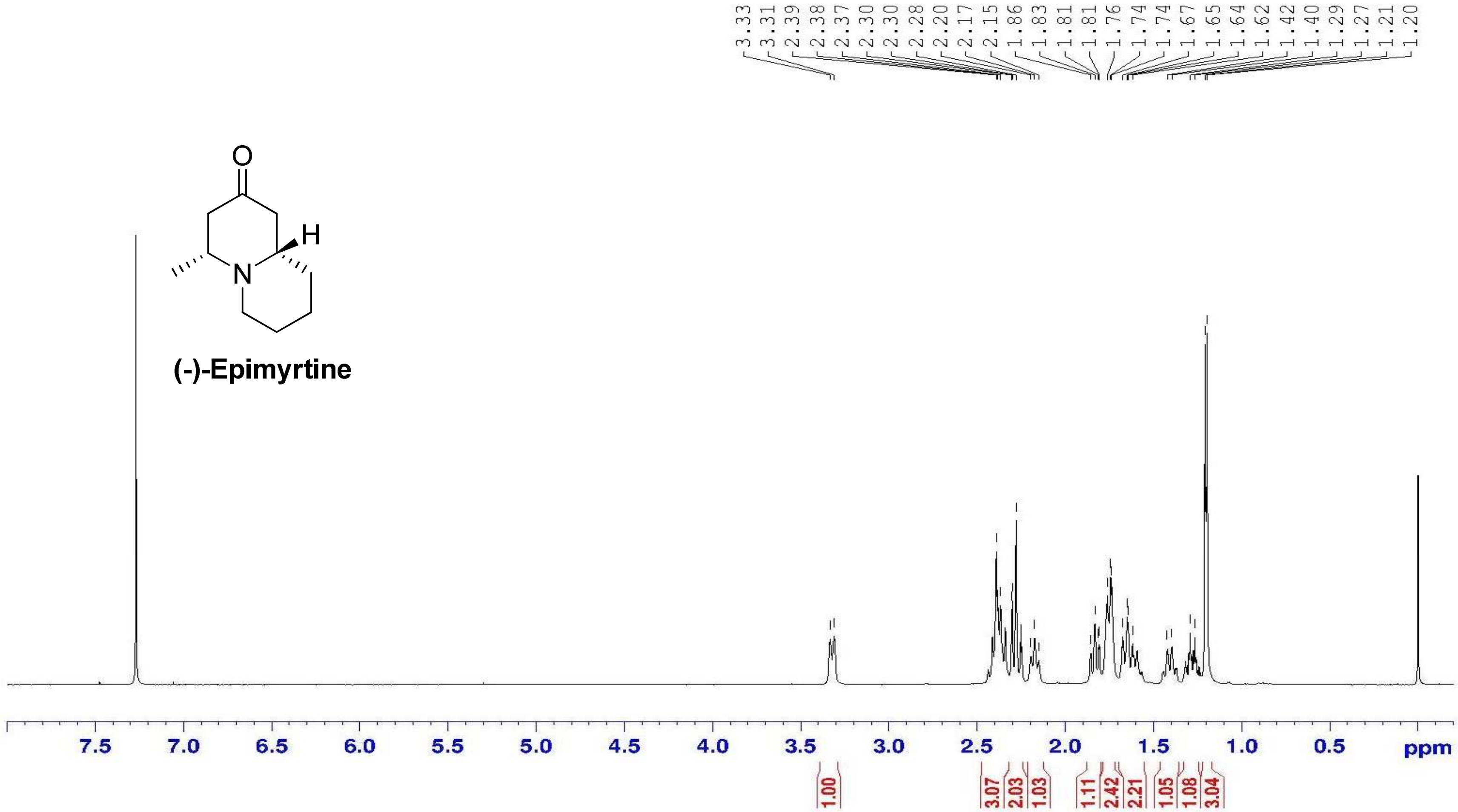
O
H
N
(-)-Epimyrtine
3.33
3.31
2.39
2.38
2.37
2.30
2.30
2.28
2.20
2.17
2.15
1.86
1.83
1.81
1.81
1.76
1.74
1.74
1.67
1.65
1.64
1.62
1.42
1.40
1.29
1.27
1.21
1.20
7.5
7.0
6.5
6.0
5.5
5.0
4.5
4.0
3.5
3.0
2.5
2.0
1.5
1.0
0.5
ppm
1.00
3.07
2.03
1.03
1.11
2.42
2.21
1.05
1.08
3.04

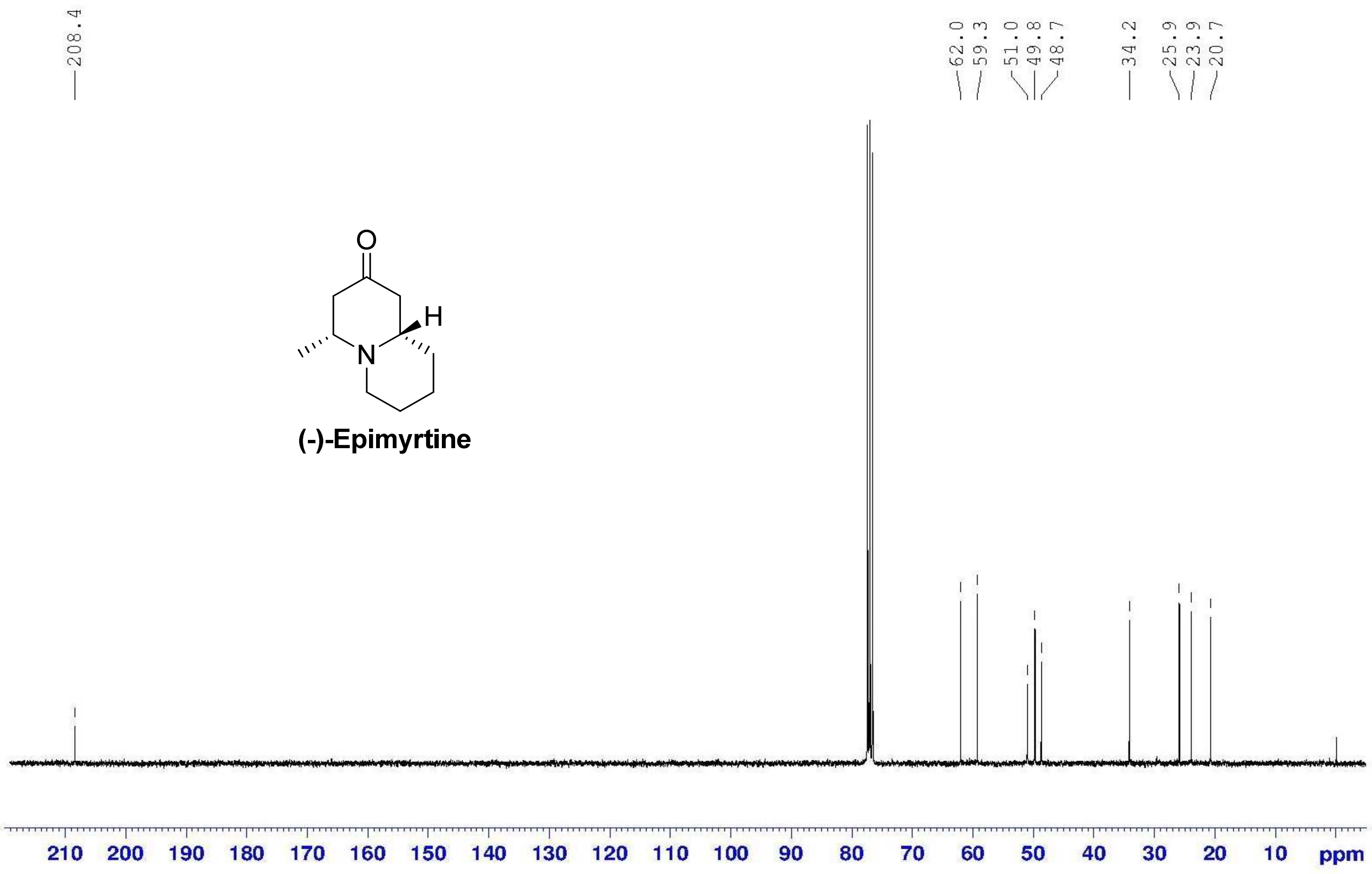
208.4
62.0
59.3
51.0
49.8
48.7
34.2
25.9
23.9
20.7
O
H
N
(-)-Epimyrtine
210
200
190
180
170
160
150
140
130
120
110
100
90
80
70
60
50
40
30
20
10
ppm
